# Genome-wide association study-driven identification of thrombomodulin and factor V as the best biomarker combination for deep vein thrombosis

**DOI:** 10.1186/s44342-025-00047-2

**Published:** 2025-05-15

**Authors:** Usi Sukorini, Gisca Ajeng Widya Ninggar, Mohammad Hendra Setia Lesmana, Lalu Irham, Wirawan Adikusuma, Hegaria Rahmawati, Nur Imma Fatimah Harahap, Chiou-Feng Lin, Rahmat Dani Satria

**Affiliations:** 1 Department of Clinical Pathology and Laboratory Medicine, Faculty of Medicine, Public Health and Nursing, Universitas Gadjah Mada, Yogyakarta, 55281 Indonesia; 2 Integrated Laboratory Installation, Dr. Sardjito General Hospital, Yogyakarta, 55281 Indonesia; 3 Department of Nursing, Faculty of Medicine, Public Health and Nursing, Universitas Gadjah Mada, Yogyakarta, 55281 Indonesia; 4 Faculty of Pharmacy, Ahmad Dahlan University, Yogyakarta, Indonesia; 5 Research Center for Computing, Research Organization for Electronics and Informatics, National Research and Innovation Agency (BRIN, Cibinong, 16911 Indonesia; 6 Department of Pharmacy, University of Muhammadiyah Mataram, Mataram, 83127 Indonesia; 7 PKU Muhammadiyah Hospital, Gombong, Kebumen, Central Java Indonesia; 8 Faculty of Medicine, Universitas ‘Aisyiyah Yogyakarta, Yogyakarta, Indonesia; 9 Integrated Clinical Laboratory Installation, Universitas Gadjah Mada Academic Hospital, Yogyakarta, 55291 Indonesia; 10 Department of Microbiology and Immunology, School of Medicine, College of Medicine, Taipei Medical University, Taipei, Taiwan; 11 Graduate Institute of Medical Sciences, Taipei Medical University, Taipei, Taiwan

**Keywords:** Deep vein thrombosis, Biomarkers, Genome-wide association studies, Single nucleotide polymorphisms, Thrombomodulin, Factor V, THBD, F5

## Abstract

Deep vein thrombosis (DVT) is a clinically significant condition characterized by the formation of thrombi in deep venous structures, leading to high morbidity and potential mortality. Identifying reliable biomarkers for DVT risk prediction remains challenging due to the intricate genetic and molecular mechanisms underlying the disease. This study aims to investigate the best biomarker for DVT. Our study utilized genome-wide association studies (GWAS) findings coupled with a functional annotation scoring system to identify and prioritize genetic markers with strong associations to DVT. Furthermore, gene expression levels were analyzed to determine the most promising genetic markers. Several databases were utilized, including the GWAS Catalog, HaploReg 4.2, WebGestalt, Enrichr, and the GTEx Portal. Through the comprehensive analysis, we found 5 potential biomarkers and highlighted thrombomodulin (THBD) and Factor V (F5) as the best blood-based biomarkers. THBD and F5 genes were selected based on their elevated expression levels in blood and the presence of eQTLs. Functionally, THBD modulates coagulation via protein C activation, while F5 is pivotal in thrombin formation and clot stabilization, underscoring their mechanistic relevance to DVT pathogenesis, and rendering them suitable for non-invasive clinical assessment. Our findings emphasize the potential of genetic biomarkers to transform DVT risk assessment and support advancements in precision medicine for thrombotic disorders.

**Supplementary Information:**

The online version contains supplementary material available at 10.1186/s44342-025-00047-2.

## Introduction

Deep vein thrombosis (DVT) or venous thromboembolism is a complex disorder involving the formation of blood clots in deep veins and is a leading cause of morbidity and mortality worldwide. It often precedes severe complications such as pulmonary embolism (PE) and poses significant healthcare burdens globally [1, 2]. The incidence was estimated approximately to be more than 10 million cases per year worldwide and increased with age [3, 4]. Over 1.2 million people were reported to have venous thromboembolism in the USA in 2016, and 40% presented as pulmonary embolism. The Mortality rate of a pulmonary embolism at 1 year is 19,6%, which unfortunately has been unchanged since 1999 [5].

A wide range of biomarkers has been explored for the diagnosis and prognosis of DVT. These include traditional coagulation markers such as prothrombin time (PT), activated partial thromboplastin time (APTT), and coagulation factors, as well as markers of coagulation activation like prothrombin fragment 1 + 2 (F1 + 2), thrombin-antithrombin complexes (TAT), and fibrin degradation products [6, 7]. Markers of fibrinolysis, such as plasminogen activator inhibitor-1 (PAI-1) and tissue plasminogen activator (t-PA), along with fibrinolysis activation markers like D-dimer and fibrin degradation products (FDP), provide insight into the dynamic balance between clot formation and breakdown [8]. Inhibitors such as antithrombin and other regulatory proteins further contribute to the complex hemostatic system [8, 9]. Despite the abundance of biomarkers, there is still no consensus on the optimal combination of biomarkers for DVT risk assessment.

Given these considerations, this study aims to systematically identify and validate the most promising biomarkers for deep vein thrombosis (DVT). By leveraging genome-wide association study (GWAS) datasets, functional annotation scoring, and gene expression profiling, we seek to prioritize genetic markers with strong mechanistic relevance to DVT [10]. We hope that the findings from this study will contribute to advancing global health and well-being, aligning with the United Nations’ Sustainable Development Goal (SDG) 3: Good Health and Well-being, by improving the diagnosis of DVT through a biomarker-based strategy.

## Methods

### Dataset selection

**Fig. 1:**
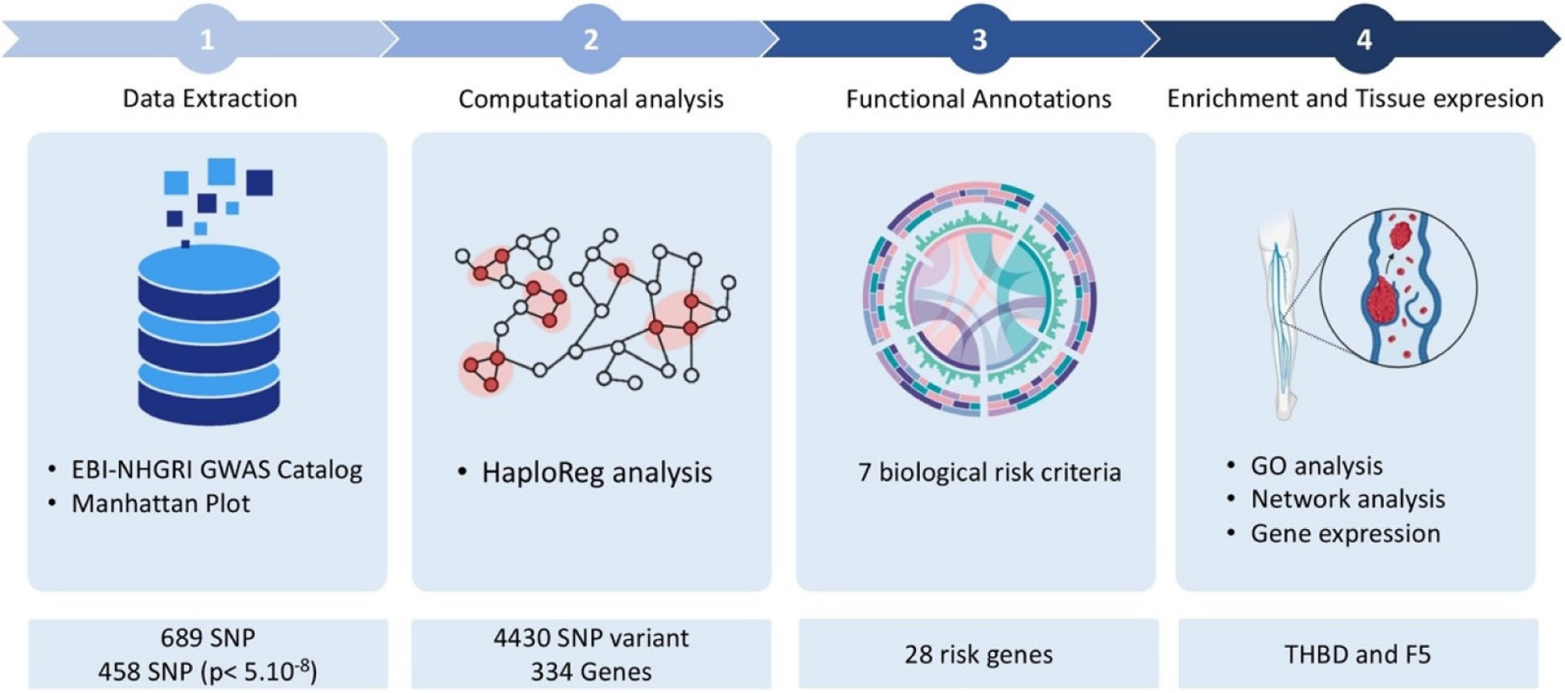
Workflow of the study for identifying blood-based biomarkers of DVT.** 1** Data Extraction: SNPs associated with DVT were extracted from the EBI-NHGRI GWAS Catalog, with a Manhattan plot used to visualize the significant SNPs. A total of 689 SNPs were identified, with 458 SNPs passing the threshold of *p* < 5.10^−^⁸. **2** Computational analysis: using HaploReg, SNP variants were analyzed, identifying 4430 SNP variants linked to 334 genes. **3** Functional Annotations: Genes were prioritized based on seven biological risk criteria to identify those with a strong association with DVT, resulting in 28 risk genes. **4** Enrichment and Tissue Expression: GO (Gene Ontology) analysis, network analysis, and tissue-specific gene expression highlighting THBD and F5 as the most promising blood-based biomarkers for DVT

Our investigation commenced by identifying genomic variants, particularly single-nucleotide polymorphisms (SNPs), that exhibit significant associations with DVT. For this purpose, we utilized data from the GWAS catalogue, applying a stringent selection criterion with a *p*-value < 5.10^−8^ to ensure the inclusion of only the strongest associated SNPs. This rigorous threshold was crucial to narrowing our focus on variants with strong statistical significance. A database search was conducted on platform accessed on October 24, 2024, from GWAS catalog (https://www.ebi.ac.uk/gwas/), to identify SNPs related to venous thromboembolism and deep vein thrombosis trait. A summary of the research workflow is shown in Fig. 1.

### Computational analysis

After extracting SNP data from the GWAS catalogue, we constructed a Manhattan plot to highlight the most significant SNPs. From this visualization, we identified the top identified genes most strongly associated with DVT. However, our focus extended beyond just these top identified genes, aiming to prioritize those with the highest biological risk potential. It is essential to recognize that not all SNPs act independently in the expression of a gene. SNPs can be influenced by neighboring variants, and there are often intricate interactions both between genes and between proteins that contribute to disease processes [10–12]. For this reason, we used functional annotation to prioritize genes, ensuring that those selected had a strong, biologically meaningful connection to DVT development.

After the initial SNP identification, we expanded our analysis by integrating additional SNPs linked to genes. To achieve this, we employed HaploReg version 4.2, a well-established tool that provides in-depth information about regulatory variants, linked genetic variants, and other relevant annotations for gene loci [13]. This tool facilitated a more comprehensive examination of the genomic landscape surrounding DVT-associated SNPs. We specifically targeted genetic data from the Asian population. This focus on a population-specific dataset allowed for a more nuanced understanding of the genetic underpinnings of DVT within this demographic, thereby improving the relevance of our findings to populations that may exhibit different genetic susceptibilities compared to other ethnic groups [14].

### Functional annotations

To enhance the accuracy and reliability of our DVT risk gene identification, we implemented a scoring system based on several criteria. Our methodology builds on a foundational approach from Okada’s study with our additional novel modifications to include the top DVT associated genes identified through the most significant SNPs from GWAS study. 10 We applied seven criteria for functional annotation: (1) prioritizing the top genes identified from GWAS-derived DVT-associated SNPs; (2) identifying missense mutations in genes with DVT-associated SNPs that exhibit strong linkage disequilibrium (r2 > 0.80); (3) examining eQTL effects in whole blood for genes carrying DVT-associated SNPs; (4) assessing involvement in biological processes; (5) identifying associations with cellular components; (6) exploring roles in molecular functions. These last three criteria are based on Gene Ontology (GO) classifications to provide a systematic framework for function assessment. For GO, we employed the WEB-based GEne SeT AnaLysis Toolkit to focus on genes with strong functional connections to DVT risk based on GO terms. We conducted an overlap analysis with genes related to primary immunodeficiency (PID) from the 2022 International Union of Immunological Societies (IUIS) list [15], enabling us to explore potential overlaps between immunological pathways and DVT-related genes. This multi-faceted approach offers valuable insights into the genetic factors underlying DVT, with a particular focus on possible immune-related contributions to disease susceptibility.

### Enrichment, network analysis, and tissue expression

We employed a hypergeometric test to assess the statistical significance of gene enrichment within our prioritized list. This test enabled us to determine whether specific genes were disproportionately represented among our DVT risk genes. A false discovery rate (FDR) threshold of 0.05 was applied to identify genes with a statistically significant overrepresentation, ensuring that our selected genes were biologically relevant and likely to contribute to DVT pathogenesis. We conducted a network analysis to further elucidate the functional roles of these genes and their associations with DVT-related pathways. We analyze gene expression profiles across a variety of tissues, providing insights into the tissue-specific expression patterns of these genes, which are critical for their potential as biomarkers.

### Statistical analysis

Statistical analyses were performed using RStudio version 2024.09.0 + 375 (RStudio, Boston, MA, USA). The haploR package (https://cran.r-project.org/web/packages/haploR/index.html ) was utilized to identify missense variants and Whole Blood eQTLs. Gene Ontology (GO) enrichment analyses, including biological processes, cellular components, and molecular functions, were performed using the WEB-based GEne SeT AnaLysis Toolkit (https://www.webgestalt.org ). Network analysis was conducted with Enrichr (https://maayanlab.cloud/enrichr-kg ) to investigate gene-disease interactions. GTEx portal (https://gtexportal.org/home/ ) was used to analyze gene tissue expression.

## Results

### Visualizing DVT-associated SNPS: GWAS Manhattan plot analysis

**Fig. 2:**
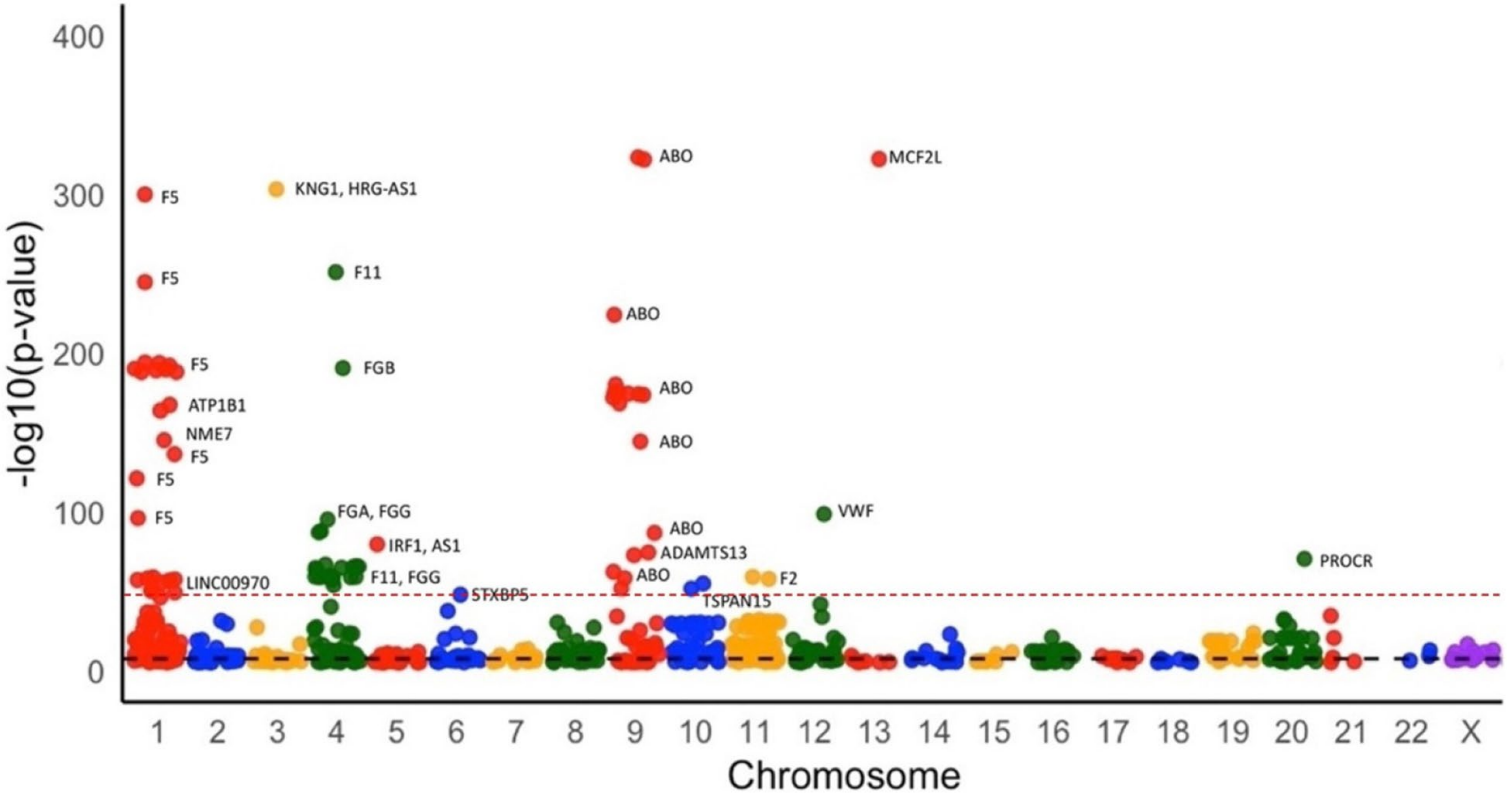
Manhattan plot of SNPs associated with DVT. 689 SNPs across chromosomes are shown with -log10 (*p*-value) on the y-axis, highlighting significant associations with DVT. Peaks on chromosomes 1, 4, 9, and 13 indicate SNPs loci that may influence DVT susceptibility. The black dashed line represents the genome-wide significance threshold (*p* = 5 × 10⁻⁸). The red dashed line indicates an additional threshold corresponding to the top 40 SNPs, based on visual prominence

The Manhattan plot illustrates the results of total 689 SNPs from a genome-wide association study (GWAS) aimed at identifying SNPs associated with DVT (Supplementary file 1). SNPs are plotted based on their chromosomal positions and association levels with DVT risk, with higher -log10 (p-value) values indicating stronger associations (Fig. 2). Notable peaks were observed at SNP loci on chromosomes 1, 4, 9, and 13 that exceed the threshold for statistical significance, suggesting their possible roles in DVT susceptibility. These areas of high association may indicate regions that contain candidate genes or regulatory elements influencing DVT development, warranting further exploration to understand the biological mechanisms involved. This analysis highlights key genomic regions that may contribute to the genetic basis of DVT, with certain SNPs showing a stronger association with the condition. The peaks on specific chromosomes may suggest genetic variations affecting pathways related to DVT pathogenesis. Identifying these SNPs provides a foundation for further studies to clarify their functions and assess their potential as biomarkers. The decision to focus on the top 40 SNPs was guided by both their visual prominence in the Manhattan plot (i.e., those appearing above the red dashed line) and the consistency of their functional annotation scores. Expanding the selection beyond the top 40 SNPs did not reveal any additional genes with a functional annotation score ≥ 3, thereby supporting the choice of 40 SNPs as a balanced and justified cut-off for further analysis.

The list showcases the SNPs with the strongest associations and their mapped genes, based on the highest peaks observed in the Manhattan plot (Table 1). Notably, the ABO gene on chromosome 9 appears frequently, indicating a potential major role in influencing DVT risk [16]. Other key genes include F5 on chromosome 1, known for its role in coagulation processes and its established link to thrombosis. Additional genes, such as ATP1B1, NME7, and FGB, are also listed, hinting at their possible involvement in DVT. This analysis reinforces the idea that DVT has a polygenic basis, with multiple genes potentially contributing to its development. The frequent identification of coagulation-related genes aligns with the known pathophysiology of DVT, while the association with the ABO gene may point to mechanisms tied to blood type and vascular health [3, 16]. Further investigation is necessary to elucidate the biological roles of these SNPs and understand how genetic variations contribute to DVT risk.

**Table 1:**
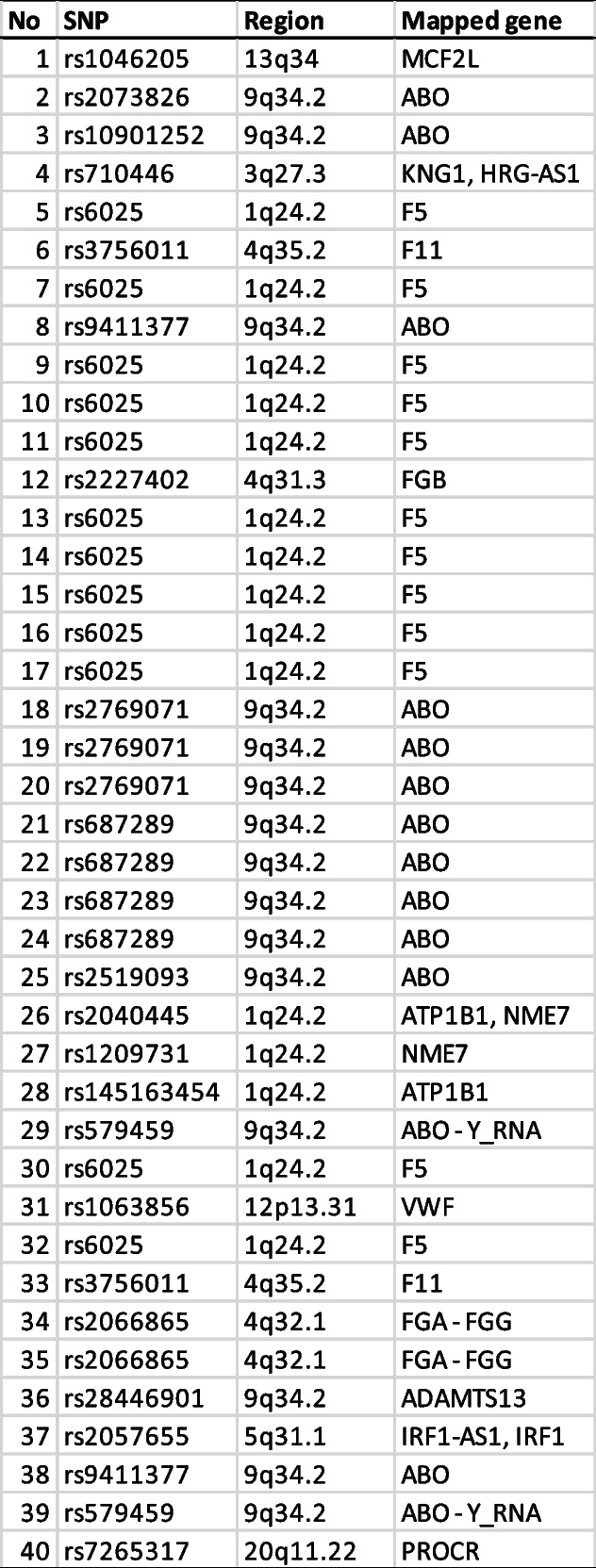
Top DVT-associated genes

This table presents top genes derived from SNPs associated DVT as identified from the Manhattan plot. Highlighted among the top genes is F5 emphasizing its potential as key biomarkers for DVT.

### Identification of genes associated with DVT

This study identified 458 SNPs from the GWAS catalog that met the inclusion criterion of *p* < 5.10^−8^ out of a total of 689 SNPs (Supplementary file 1). It is important to understand that SNPs do not always function independently in gene expression. They can be influenced by nearby SNP variants, and complex interactions frequently occur between genes and proteins, collectively contributing to disease mechanisms [17]. To ensure comprehensive coverage and minimize the possibility of missing relevant genetic variations, we expanded the initial 458 SNPs using data from the Human Genome Project via HaploReg version 4.2. This expansion applied a linkage disequilibrium threshold of *r*^2^ > 0.8 in the Asian population, enabling the inclusion of additional variants that are strongly linked to the originally identified SNPs. This process yielded 4430 SNPs, which were responsible for 334 genes associated with DVT (Supplementary file 2). These genes were subsequently selected for further analysis.

### Mapping biological risk genes in DVT: a functional annotation approach

To determine the biological roles of these 334 genes, we performed functional annotation using seven databases to prioritize these genes. This approach was based on the rationale that gene–disease associations may be influenced not only by gene expression but also by various other biological processes. The underlying assumption is that genes annotated in multiple functional annotations are more likely to play a crucial role in DVT development.

We used a minimum score criterion of 3 to determine genes considered to play a significant role in the pathology of DVT. Based on functional annotation criteria, we identified 28 biological risk genes associated with DVT (Table 2). Among the genes listed, IL6R, F5, and ABO stand out with high scores, totaling 6, 5, and 5, respectively. These scores indicate that these genes meet multiple functional criteria, suggesting a significant role in DVT development. The extensive annotation profile of IL6R highlights it as a key candidate in DVT research, likely due to its involvement in inflammatory signaling pathways that influence vascular function. Inflammation is closely associated with thrombosis, and IL6R’s role in modulating immune responses may impact both vascular health and blood clotting dynamics, which are critical factors in DVT risk [18].

**Table 2:**
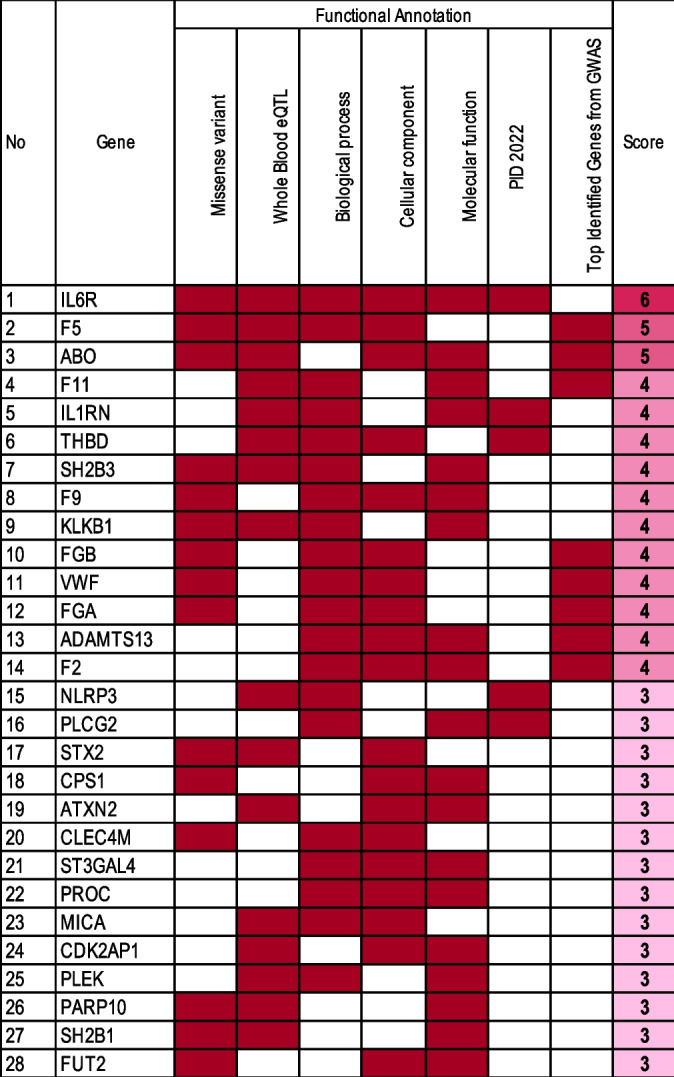
Functional annotation scores of top DVT-associated genes

This table provides a functional annotation of the top genes associated with DVT, scored based on seven criteria: missense variants, whole blood eQTL presence, involvement in biological processes, cellular components, molecular functions, overlap with primary immunodeficiency (PID) genes, and inclusion in the top identified DVT-associated genes.

Factor V (F5) and ABO also receive high scores (5 each) and are among the top DVT- associated genes based on SNP significance. This distinction highlights their strong association with DVT-related genetic variations. Factor V, known for its role in coagulation, is particularly relevant as it directly influences blood clotting pathways—a central aspect of DVT pathology [19]. Genetic variations in F5, such as the well-known Factor V Leiden mutation, have been widely studied for their impact on clotting risk, reinforcing the gene’s importance in DVT [20]. The ABO gene has been linked to DVT through blood group associations. Certain blood types are known to carry a higher risk of thrombosis, and genetic variations in ABO may contribute to this predisposition [16]. The strong annotation scores for both F5 and ABO emphasize their roles as central players in DVT pathogenesis, further justifying their prioritization in functional analysis.

Other notable genes such as F2, F9, F11, IL1RN, KLKB1, SH2B3, FGB, VWF, FGA, ADAMTS13, and THBD have scored 4, meeting several annotation criteria. These genes are often linked to pathways essential to DVT, including those governing coagulation and immune function. Supporting our findings, F9, F11, and KLKB1 are well-known for their roles in blood coagulation pathways [21, 22] Their presence in this list underscores the importance of coagulation in DVT pathophysiology. IL1RN, SH2B3, and THBD, with known functions in immune regulation and endothelial health further emphasize the role of the immune system and vascular integrity in DVT [23]. Abnormalities in these pathways can increase clotting risk and impact vascular health, key elements in DVT development.

Overall, the table effectively organizes DVT-associated genes by their functional annotations, highlighting those with the strongest potential roles in DVT pathogenesis. By using a multi-criteria approach to prioritize genes, the analysis helps pinpoint those most relevant for further study. This table, therefore, serves as a foundational step in translating genetic findings into actionable clinical insight.

### Identification coagulation-related genes

**Fig. 3:**
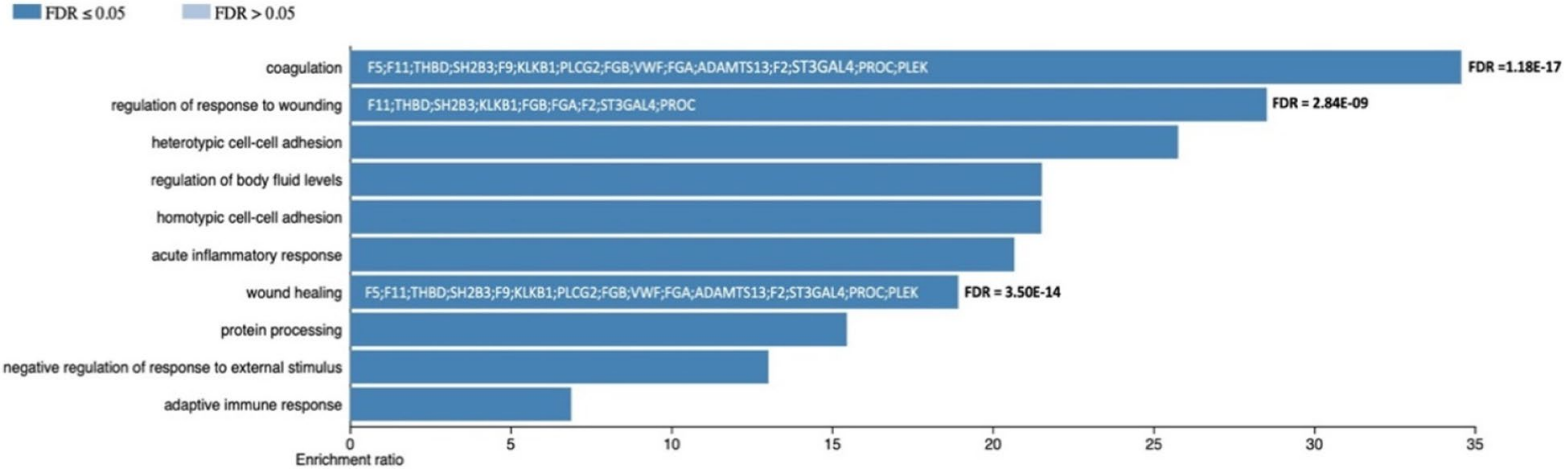
Gene ontology (GO) enrichment analysis of biological processes related to DVT-associated genes. This bar plot illustrates the enriched biological processes for genes associated with DVT, indicated by enrichment ratio and adjusted by false discovery rate (FDR). Notably, 15 genes involved in coagulation, regulation of response to wounding, and wound healing (FDR ≤ 0.05). These processes highlight the involvement of these genes in clot formation and tissue repair mechanisms, providing valuable insights into their roles in DVT pathogenesis

We performed enrichment analysis on the 28 genes that had a minimum functional annotation score of 3 to determine whether these genes play a significant role in the pathophysiology of DVT. The results confirmed this hypothesis, as the enrichment analysis highlighted their strong involvement in coagulation-related pathways, providing valuable insights into the genetic factors underlying DVT (Fig. 3). This analysis shows significant enrichment in coagulation-associated pathways, suggesting that these genes primarily influence the processes of clot formation. Pathways related to regulation of response to wounding, wound healing are notably prominent, indicating that these genes may help maintain the balance between clot-promoting and clot-preventing forces in blood vessels.

Based on the significant enrichment in coagulation, regulation of response to wounding and wound healing (Fig. 3), we identified 15 genes (*F5; F11; THBD; SH2B3; F9; KLKB1; PLCG2; FGB; VWF; FGA; ADAMTS13; F2; ST3GAL4; PROC; PLEK*) for further analysis to elucidate their potential roles in DVT pathogenesis.

The prominent role of these coagulation pathways implies that many of these genes might act as regulators or modulators of thrombin and fibrin activity, both crucial to clot formation. Disruptions in these pathways could lead to excessive clotting or inadequate clot resolution, both of which are risk factors for DVT.

### Uncovering five genetic drivers of DVT: insights from network analysis

**Fig. 4:**
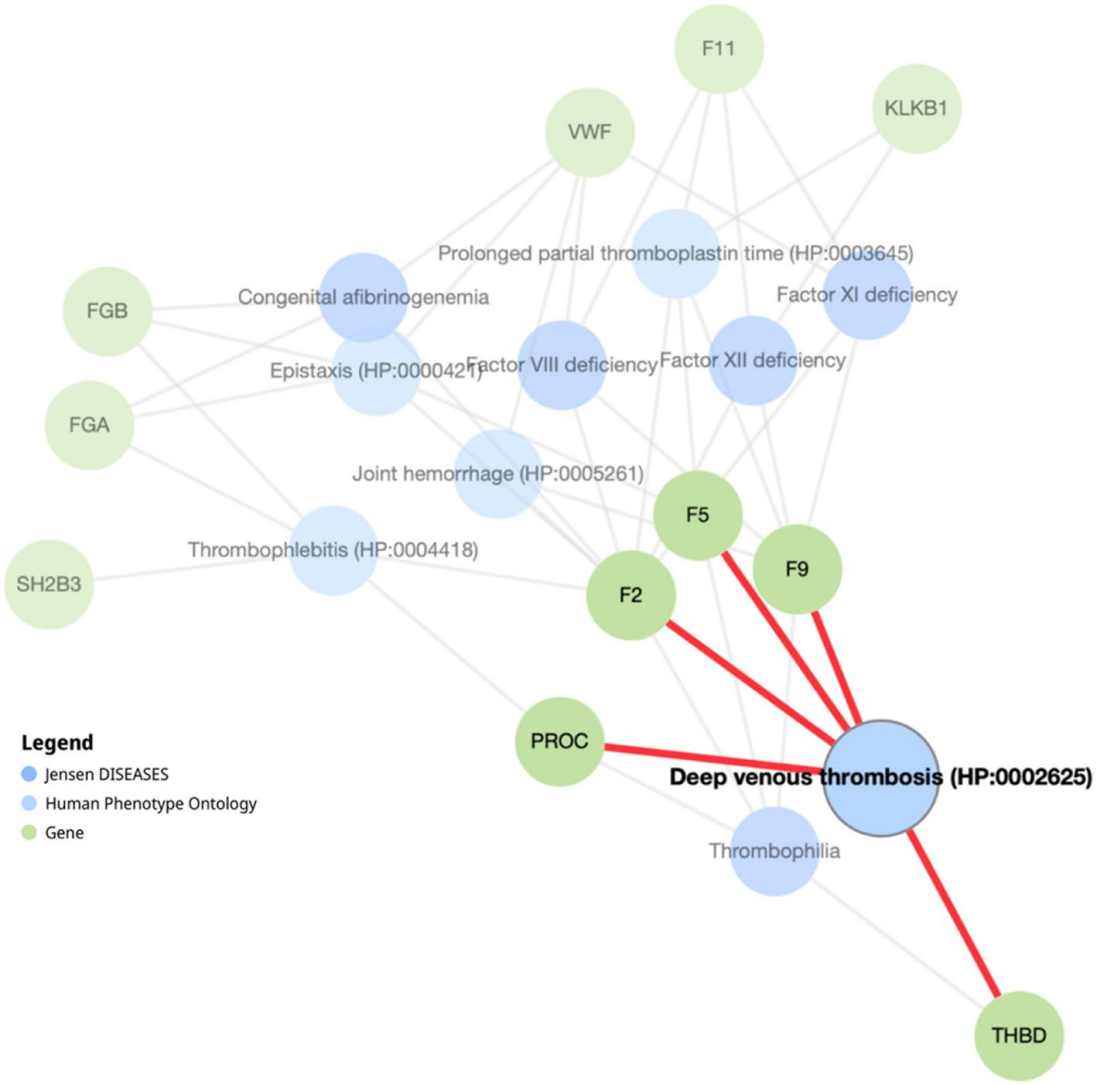
Network analysis of key genes associated with DVT and thrombophilia. This network illustrates the relationships between genes (green nodes), human phenotypes (blue nodes), and disease ontologies linked to DVT and thrombophilia. The highlighted genes *F5*, *F2*, *F9*, *PROC*, and *THBD* show direct connections (red lines) to DVT, underscoring their central role in coagulation pathways and thrombotic risk. This network highlights gene-phenotype and gene-disease associations that may contribute to the genetic susceptibility and pathogenesis of thrombotic disorders

To investigate the connections between these genes and DVT-related phenotypes, we selected 15 genes from the previous enrichment analysis to perform network analysis based on the Jensen Disease and Human Phenotype Ontology databases. This analysis reveals five key genes—*F2, F5, PROC, F9,* and *THBD*—with significant links to DVT, as illustrated in Fig. 4. Exploring genetic relationships and associated phenotypes, this provides a clearer understanding of the molecular basis of DVT. The network diagrams show specific interactions between these genes and pathways related to coagulation, providing insight into their functions in thrombotic conditions.

Each of these 5 genes shows a meaningful association with DVT and thrombophilia phenotypes, highlighting their critical roles in thrombosis. F2 and F5 are fundamental to blood clotting and are directly linked to processes associated with DVT, underlining their importance in clot stability and formation [19, 24]. PROC serves as a natural anticoagulant by deactivating certain clotting factors, and alterations in this gene have been associated with a higher risk of thrombosis [25]. F9, essential in the intrinsic coagulation pathway, plays a vital role in thrombin generation [26]. Thrombomodulin (THBD), known for its dual function in promoting anticoagulation and preserving vascular health, appears linked to DVT, suggesting that disruptions in THBD could undermine these protective roles and increase the likelihood of thrombosis [27].

Identifying these genes as central elements in the DVT network highlights their value as potential biomarkers. Given their strong associations with DVT and coagulation pathways, these genes are likely valuable indicators for evaluating DVT risk and understanding broader coagulation-related issues. As biomarkers, they could play a vital role in early detection, risk evaluation, and tracking the progression or recurrence of DVT, contributing to better management and outcomes for individuals with thrombotic disorders.

### F2, F5, F9, PROC, and THBD gene expression across different tissues: focus on liver and whole blood

**Fig. 5:**
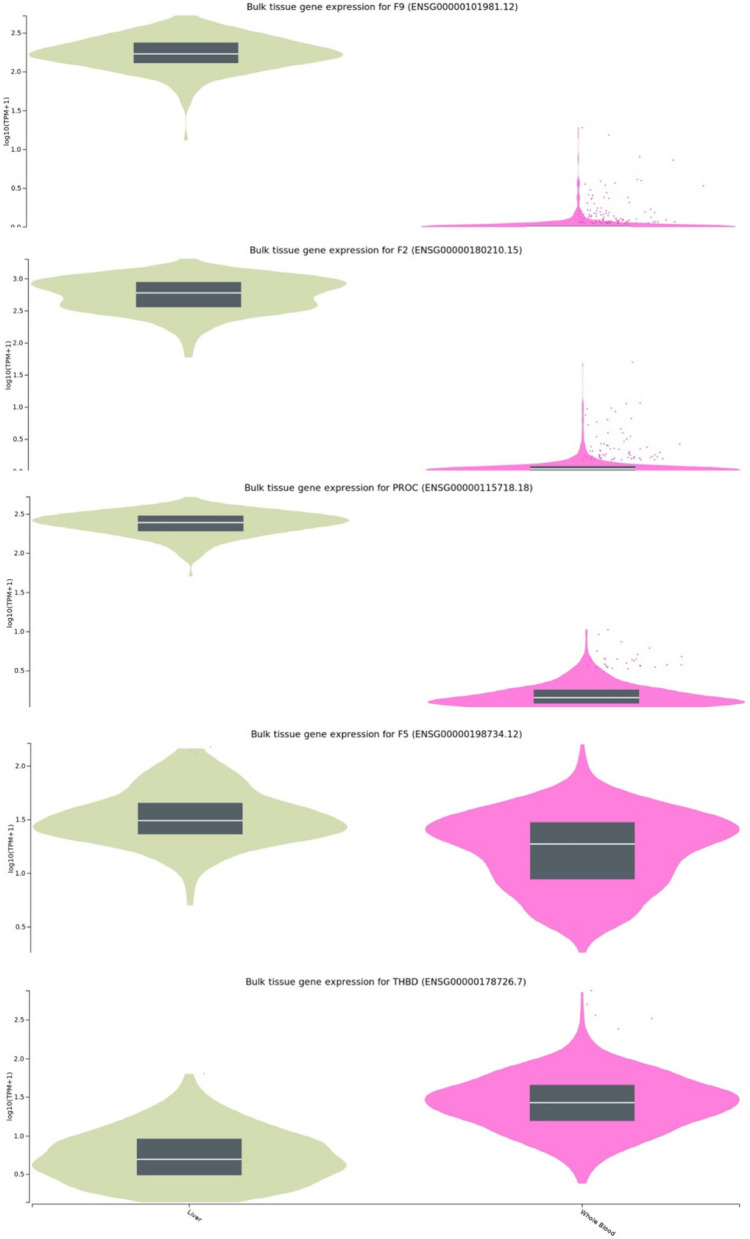
Gene expression profiles of DVT-associated genes in liver and whole blood. Violin plots representing the bulk tissue gene expression of five key genes associated with DVT: F9, F2, PROC, F5, and THBD. Each plot shows expression levels in liver (green) and whole blood (pink) tissues, as analyzed from GTEx data. THBD and F5 exhibit significant expression in whole blood, suggesting their potential as blood-based biomarkers for DVT. In contrast, F2, F9, and PROC show higher expression levels in the liver, indicating a more organ-specific role. The broad expression of THBD and F5 in whole blood highlights their suitability biomarker of DVT

Based on the gene expression patterns displayed for Prothrombin/Factor II (F2), Factor V (F5), Factor IX (F9), Protein C (PROC), and Thrombomodulin (THBD) in the liver and whole blood, the selection of effective biomarkers for deep DVT can be refined (Fig. 5). An ideal biomarker should not only be relevant to DVT but also detectable in a sample that is easy to collect, such as blood. From the visual data, THBD and F5 emerge as top candidates due to their substantial expression in vein (whole blood) tissue, as shown by the broad distribution seen in the data. This high level of expression indicates that these genes are actively involved in coagulation processes in the bloodstream, making them well-suited for blood-based detection relevant to DVT.

In contrast, F2, F9, and PROC exhibit higher expression predominantly in liver tissue, suggesting a more localized function in that organ. However, it is also noteworthy that the expression of F2, F9, and PROC in blood varies among normal individuals, even though their average levels are lower than in liver tissue. This variation suggests that, while these genes are not expressed consistently at high levels in the blood, some degree of activity does occur in the bloodstream. Yet, due to the variability and generally lower expression in blood compared to the liver, F2, F9, and PROC may be less practical as direct blood-based biomarkers for DVT.

As shown in Table 2, there are expression quantitative trait loci (eQTLs) for THBD and F5 in whole blood, reinforcing their applicability as blood-based biomarkers for DVT. In contrast, no eQTLs are observed in blood for PROC, F2, and F9, suggesting that these genes may not be as effective for direct detection in blood. This distinction further supports THBD and F5 as promising candidates for blood-based DVT biomarkers, as their genetic variants actively influence expression levels in the blood, providing a more practical basis for clinical testing.

Therefore, THBD and F5 stand out as practical biomarkers due to their robust expression in the blood, enabling easier, minimally invasive sampling and testing. Monitoring THBD and F5 levels in blood could provide a reliable approach for assessing DVT risk, offering valuable insights for clinical diagnostics and risk management in thrombotic conditions.

## Discussion

This study contributes to the understanding of genetic factors associated with DVT by identifying key biomarkers that could enhance both risk evaluation and clinical diagnostics. Through the integration of network analysis and functional annotation, we highlighted five significant genes: F2, F5, PROC, F9, and THBD. Among these, THBD and F5 stood out as particularly suitable blood-based biomarkers. Their substantial expression levels in blood (Fig. 5), with the presence of expression quantitative trait loci (eQTLs) in whole blood (Table 2), make them viable candidates for clinical use. The accessibility of blood samples supports non-invasive, repeatable testing, making THBD and F5 preferable for clinical application over other tissue-specific markers.

The functional roles of THBD and F5 emphasize their importance as biomarkers and elucidate their mechanisms in DVT pathophysiology. Thrombomodulin, expressed on endothelial cells, binds to thrombin, facilitating the activation of protein C (PC) into its activated form (aPC). Activated protein C acts as a natural anticoagulant by inactivating Factor Va, the active form of Factor V, thus modulating thrombin generation and preventing excessive clotting [19, 28]. This anticoagulant pathway is vital for maintaining a balance in coagulation, safeguarding against excessive clot formation that could lead to thrombosis. Therefore, genetic variants affecting THBD expression or function may disrupt this equilibrium, raising thrombotic risk [27, 29].

Factor V plays a central role in clot formation and stability. Variants in F5, such as the Factor V Leiden mutation, are associated with increased thrombotic risk due to their resistance to degradation by activated protein C [30, 31]. The functional interplay between THBD and F5 is crucial, as thrombomodulin-mediated protein C activation provides a natural mechanism to counteract Factor V’s pro-coagulant effects [27]. Disruptions in this regulatory process could result in a pro-thrombotic state, underlining the relevance of both THBD and F5 in DVT pathogenesis and their suitability as biomarkers.

In contrast, while F2, PROC, and F9 are essential components of the coagulation cascade, their high expression levels in liver tissue and lack of blood-based eQTLs make them less suitable as blood biomarkers for DVT [26, 32]. Despite their significant functions in coagulation, these genes may be more applicable for liver-specific analyses rather than blood-based diagnostics. The observed variability in their blood expression among healthy individuals further limits their reliability as consistent blood biomarkers, as such fluctuations may not correlate directly with thrombotic risk (Fig. 5).

The polygenic nature of DVT is affirmed by this study, with multiple genes contributing to coagulation and thrombotic risk across various pathways. The combined use of GWAS data and functional annotation analysis allowed for the selection of biomarkers that are not only biologically significant but also practical in a clinical setting. The presence of eQTLs for THBD and F5 in blood highlights their potential application in an ELISA-based assay to detect their protein levels in plasma. Such a diagnostic tool would offer a non-invasive, cost-effective, and practical approach for early DVT detection in hospital settings.

The limitation of this study is that the potential biomarkers, F5 and THBD, may represent only a small fraction of the total genetic variation contributing to DVT. While this approach aimed to prioritize genes based on functional annotation, it is possible that other relevant genes or regulatory regions were not captured. Therefore, future studies involving more comprehensive and integrative analyses are warranted to validate the utility of THBD and F5 as biomarkers for DVT across diverse populations. Such studies should evaluate how their expression interacts with established risk factors, including age, immobility, and metabolic conditions. By integrating THBD and F5 into clinical risk models, we may improve diagnostic precision and enable more personalized approaches to DVT management.

## Conclusion

In conclusion, this study identifies THBD and F5 as promising blood-based biomarkers for assessing DVT risk. Their strong association with DVT-related pathways, high blood expression levels, and the presence of eQTLs support their clinical applicability for non-invasive testing. This research lays a foundation for future work to bridge genomic findings with practical clinical applications in DVT.

## Supplementary Information

Supplementary Material 1. The identified 458 SNPs from the GWAS catalog that met the inclusion criterion of *p* < 5.10^−8^ out of a total of 689 SNPs

Supplementary Material 2. The process yielded 4430 SNPs, which were responsible for 334 genes associated with DVT.

## Data Availability

No datasets were generated or analysed during the current study.
